# Autoimmune Conversion of Hashimoto’s Thyroiditis to Graves’ Disease With Immune Pancytopenia in a Patient With Seropositive Rheumatoid Arthritis: A Diagnostic and Therapeutic Conundrum

**DOI:** 10.7759/cureus.116714

**Published:** 2026-09-22

**Authors:** Sangita D Kamath, Bijaya Jha, Vijay Kota, Prashant U Kaduskar

**Affiliations:** 1 General Medicine, Tata Main Hospital, Jamshedpur, IND; 2 Endocrinology, Tata Main Hospital, Jamshedpur, IND

**Keywords:** autoimmune switch, carbimazole, graves’ disease, hashimoto’s thyroiditis, pancytopenia, rheumatoid arthritis, thyrotoxicosis

## Abstract

Autoimmune conversion of Hashimoto’s thyroiditis (HT) to Graves’ disease (GD) is an uncommon phenomenon. The coexistence of GD, seropositive rheumatoid arthritis (RA), and pancytopenia presents a rare diagnostic and therapeutic challenge, particularly when antithyroid drug (ATD) therapy is contemplated in the presence of pre-existing cytopenias.

A 66-year-old woman with a four-year history of hypothyroidism on levothyroxine replacement and long-standing seropositive RA presented with generalized weakness, altered bowel habits, and insomnia. Serial thyroid function tests (TFTs) had demonstrated progressive suppression of thyroid-stimulating hormone (TSH), necessitating dose reduction and eventual discontinuation of levothyroxine. Evaluation revealed thyrotoxicosis with elevated free thyroxine (FT4), free triiodothyronine (FT3), raised antithyroid peroxidase and TSH receptor antibodies, diffuse goiter with increased vascularity on Doppler ultrasonography, and diffusely increased uptake on thyroid scintigraphy, confirming Graves’ disease. Concurrent investigations demonstrated pancytopenia with elevated liver enzymes and mild splenomegaly. Given the presence of seropositive rheumatoid arthritis and the exclusion of alternative causes of pancytopenia, an immune-mediated mechanism leading to bone marrow suppression was suspected. After treatment with granulocyte colony-stimulating factor, carbimazole was cautiously initiated with close hematological monitoring. The patient showed progressive clinical and hematological improvement.

This case highlights the rare transition from Hashimoto’s thyroiditis to Graves’ disease in a patient with seropositive RA and immune-mediated pancytopenia. It underscores the importance of considering autoimmune thyroid conversion in previously hypothyroid patients with persistent TSH suppression and demonstrates that carefully monitored antithyroid therapy can be safely administered despite pre-existing pancytopenia within a multidisciplinary care framework.

## Introduction

Autoimmune thyroid disease comprises Hashimoto’s thyroiditis (HT) and Graves’ disease (GD), two disorders at opposite ends of the autoimmune thyroid spectrum. Although both conditions share common pathogenic mechanisms, conversion from HT to GD is a rare clinical event [1]. Such autoimmune thyroid disorders frequently coexist with other autoimmune conditions, particularly rheumatoid arthritis (RA), reflecting a common underlying autoimmune predisposition [2]. When multiple autoimmune conditions coexist, establishing the cause of systemic manifestations can be challenging and may significantly influence therapeutic decisions. We report a rare case of autoimmune conversion from HT to GD in a patient with seropositive RA and pancytopenia, highlighting the diagnostic and therapeutic complexities of management. The coexistence of severe pancytopenia and thyrotoxicosis presented a unique clinical challenge because the need for treatment had to be balanced against concerns regarding the etiology of the cytopenias and the potential effects of therapeutic interventions on blood cell counts.

## Case presentation

A 66-year-old woman presented with complaints of generalized weakness and intermittent episodes of semi-solid stools, without blood or mucus, for the preceding three months. She also reported poor sleep quality and inadequate nocturnal sleep over the past two months. She had been diagnosed with hypothyroidism four years earlier and was initially treated with oral levothyroxine 75 µg daily. Two years after the initiation of therapy, her serum thyroid-stimulating hormone (TSH) level was found to be suppressed at 0.1 IU/dL, prompting a reduction in the levothyroxine dose to 50 µg daily. Six months later, repeat thyroid function tests (TFTs) revealed further suppression of TSH to 0.01 IU/dL, following which levothyroxine therapy was discontinued, considering the possibility of an overdose. The patient was also a known case of seropositive rheumatoid arthritis (RA), detected six years earlier, and had been receiving oral methotrexate 10 mg weekly along with folic acid 5 mg twice weekly. However, she had self-discontinued these medications approximately six months before presentation. She was not a smoker and did not have other addictions. There was no history of diabetes, hypertension, cardiac disorder, or chronic renal failure.

On examination, the patient appeared lean and had mild pallor and thin, papery, shiny skin. There was no evidence of icterus, clubbing, lymphadenopathy, or pedal edema. Fixed deformities of RA were noted in the small joints of both hands and feet. There was no evidence of active synovitis. Examination of the neck revealed a small, soft goiter. There were no eye signs of thyrotoxicosis. Her vital signs were as follows: temperature, normal (afebrile); pulse rate, 112 beats/minute; blood pressure, 126/82 mmHg; respiratory rate, 18 breaths/minute; and oxygen saturation, 98% on ambient air. Systemic examination, including cardiovascular, respiratory, abdominal, and neurological evaluation, was unremarkable. Her initial blood investigations on admission are as shown in Table 1.

**Table 1:** Laboratory investigations on admission ALP: alkaline phosphatase, ALT: alanine transaminase, ANA: antinuclear antibody, anti-CCP Ab: anti-cyclic citrullinated antibody, AST: aspartate transaminase, BNP: brain natriuretic peptide, ELISA: enzyme-linked immunosorbent assay, ESR: erythrocyte sedimentation rate, HBsAg: hepatitis B surface antigen, HCV: hepatitis C virus, HIV: human immunodeficiency virus, IFA: indirect immunofluorescence, RA: rheumatoid arthritis factor The normal ranges shown are adult age-adjusted reference ranges applicable to the patient’s age. Minor variations may occur between laboratories depending on methodology and institutional standards.

Investigations	Values	Normal range
Hemoglobin (g/dL)	9.2	11.5-15.5
Total leucocyte count (cells mm^3^)	1,740	4,000-11,000
Differential count	34% neutrophils, 45% lymphocytes, 10% eosinophils	-
MCV (fL)	94.6	80-100
Platelet count (/mm^3^)	86,600	150,000-450,000
Total serum proteins (g/dL)	6.6	6.6-8.3
Serum albumin (g/dL)	2.4	3.5-5.2
Serum globulin (g/dL)	4.2	2.5-3.5
Serum creatinine (mg/dL)	0.87	0.5-1.5
Total bilirubin (mg/dL)	0.98	0.2-1
ALT	62.5	5-40
AST	144.5	5-45
ALP	236.5	35-125
ESR (Westergren) (mm/hr)	24	0-30
C-reactive protein (mg/dL)	0.37	0.5-1.5
BNP (pg/mL)	84	<100
RA factor (IU/mL)	149.2	<14
Anti-CCP Ab (U/mL)	80	<20
ANA (IFA)	Negative	-
HBsAg (ELISA)	Non-reactive	-
HCV ab	Non-reactive	-
HIV l and 2	Non-reactive	

Her thyroid function tests (TFTs) on admission revealed thyroid-stimulating hormone (TSH) of 0.01 milli-international units per liter (mIU/L) (normal range: 0.5-5 mIU/L), total thyroxine (T4) of 19.1 µg/dL (normal range: 5.4-11.5 µg/dL), free T4 of 2.9 ng/mL (normal range: 0.56-1.5 ng/mL), free T3 of 4.4 picogram/mL (normal range: 2.5-3.9 picogram/mL), anti-thyroperoxidase (TPO) antibody of 867.0 IU/mL (normal range: 3-40 IU/mL), and anti-thyroglobulin antibody of 397.8 ng/mL (normal range: 3-40 ng/mL). High-resolution ultrasonography (HRUSG) (Figure 1) with Doppler evaluation of the thyroid demonstrated enlargement of both lobes and isthmus, with heterogeneous echotexture and increased vascularity (thyroid inferno), findings suggestive of diffuse thyroid disease.

**Figure 1:**
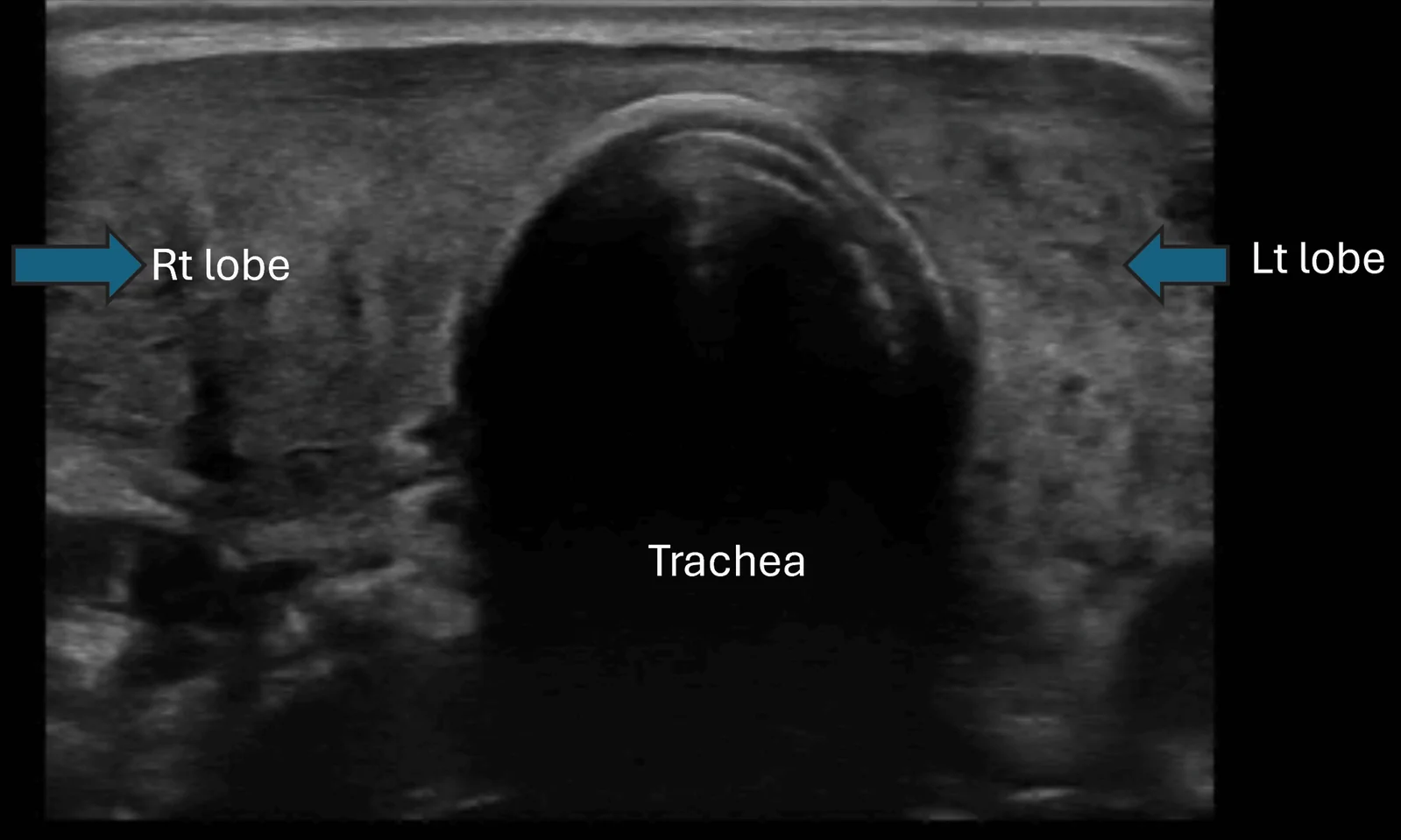
HRUSG of the thyroid showing enlargement of both lobes with heterogeneous echotexture HRUSG: high-resolution ultrasonography

In view of the clinical suspicion of thyrotoxicosis, a thyroid scintigraphy was performed using 5 millicuries of technetium-99m (Tc-99m) pertechnetate. The scan revealed diffusely increased radiotracer uptake, with a total uptake of 7.8% (normal range: 0.4%-4.0%), consistent with hyperthyroidism (Figure 2).

**Figure 2:**
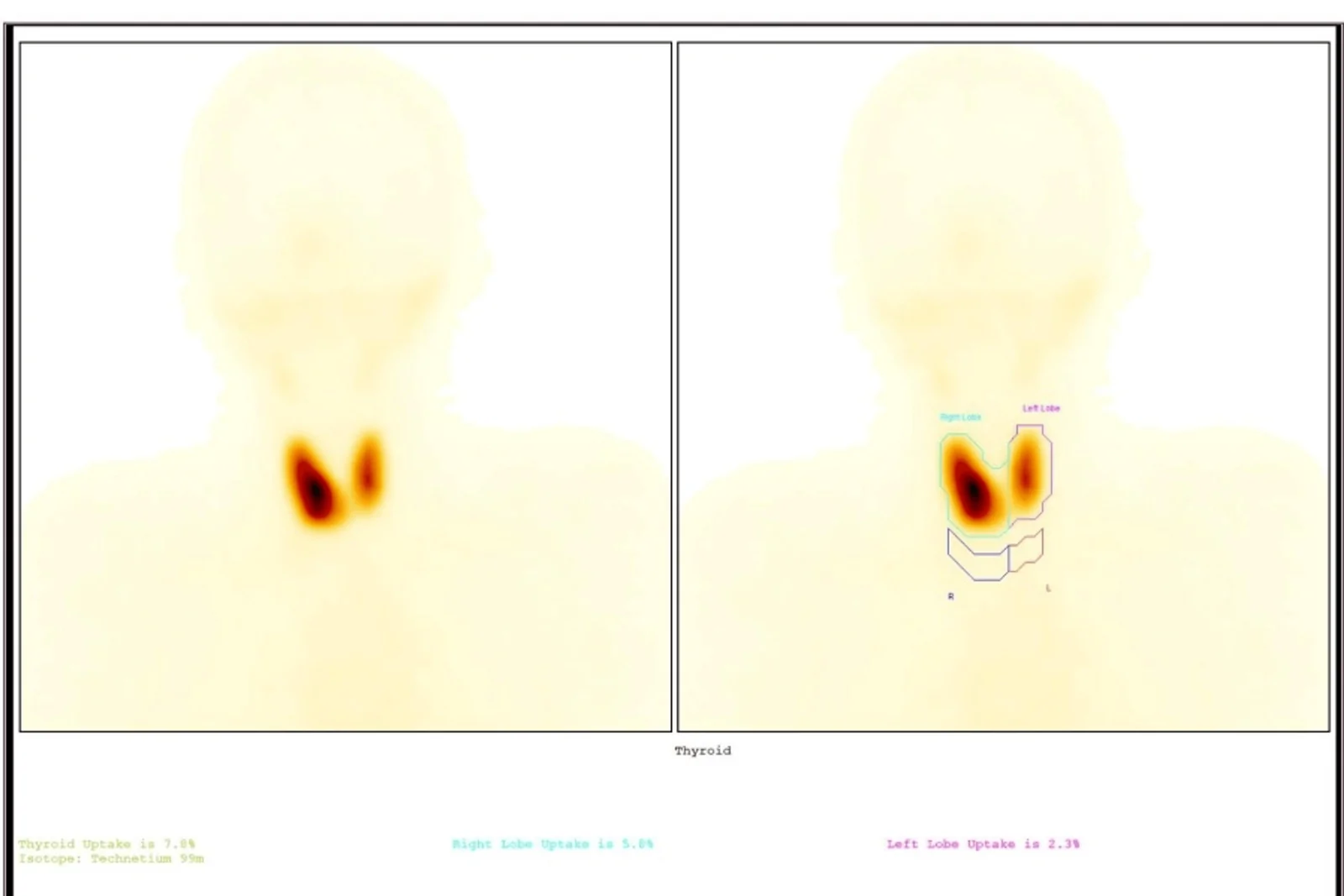
Tc-99m pertechnetate thyroid scintigraphy showing diffuse increased uptake Tc-99m: technetium-99m

To determine the etiology of hyperthyroidism, thyroid-stimulating hormone receptor antibody (TRAb) levels were measured and found to be markedly elevated at 11.6 (normal range: <1.75 IU/L). Based on the clinical, biochemical, and imaging findings, a diagnosis of thyrotoxicosis secondary to Graves’ disease was established.

Further, hematological evaluation for pancytopenia revealed normal iron studies, serum folic acid, and vitamin B12 levels as shown in Table 2.

**Table 2:** Anemia profile of the patient LDH: lactate dehydrogenase, TIBC: total iron-binding capacity

Investigations	Values	Normal range
Serum iron (mg/dL)	60.2	37-145
TIBC (mcg/dL)	184	149-492
Transferrin saturation (%)	36.9	15-50
Serum ferritin (ng/mL)	260.9	11-306.8
Serum B12 (pg/mL)	467	200-900
Serum folic acid (ng/mL)	6.2	2-10
Reticulocyte index (%)	1.2	1.6-12.1
Sickling test	Negative	-
LDH (U/L)	168	140-280

Peripheral blood smear examination showed normochromic, normocytic anemia, while the direct Coombs test was negative. Ultrasonography of the abdomen and pelvis demonstrated grade I fatty liver. Contrast-enhanced computed tomography (CECT) of the abdomen revealed normal hepatic echotexture, a portal vein of normal caliber, absence of portosystemic collaterals, and mild splenomegaly, with the spleen measuring 13.5 cm in its longest axis.

Retrieval of records of previous complete blood counts during methotrexate therapy demonstrated a gradual decline in hematological parameters without overt pancytopenia. Notably, hematological parameters continued to deteriorate following methotrexate discontinuation, with the lowest counts occurring approximately six months after withdrawal, as shown in Table 3.

**Table 3:** Serial hematological counts prior to hospital admission MTX: methotrexate, WBC: white blood cell count

Investigations	July 2025 (on MTX)	October 2025 (on MTX)	June 2026 (off MTX for 6 months)
Hemoglobin (g/dL)	10.4	10	9.6
Total WBC (/cu mm^3^)	6,780	4,970	3,000
Neutrophil (%)	84	69	60
Lymphocytes (%)	16	26	28
Monocytes (%)	0	4	6
Platelet count (/cu mm^3^)	143,200	130,100	109,000

Bone marrow aspiration and trephine biopsy were undertaken for further evaluation. Bone marrow examination demonstrated a hypocellular marrow with trilineage hematopoietic suppression. There was no evidence of active hemophagocytosis, granulomatous inflammation, lymphoma, or significant dysplasia. A few large reactive lymphocytes were noted. Fluorescence in situ hybridization (FISH) myelodysplastic syndrome (MDS) panel showed absence of deletions of 5q, 7q, and 20q chromosomes and trisomy of chromosome 8. Given the coexistence of two autoimmune disorders, an immune-mediated bone marrow suppression was considered the most likely etiology.

The coexistence of pancytopenia and deranged liver function tests posed a significant therapeutic dilemma in the initiation of antithyroid drugs (ATDs). To address the severe cytopenias, the patient received three doses of human granulocyte colony-stimulating factor (G-CSF) 300 mcg administered subcutaneously once daily for three days. Subsequent hematological assessment revealed a marked improvement in her total leukocyte count (TLC), which rose to 5,060 cells/mm³ with 56% neutrophils. In view of this encouraging response, oral carbimazole was commenced at a dose of 10 mg twice daily, along with long-acting propranolol 40 mg once daily.

Despite the initial improvement, her TLC declined again to 2,840 cells/mm³ with 45% neutrophils on the fourth day of therapy. The persistence of mildly elevated liver enzymes precluded the use of propylthiouracil as an alternative antithyroid agent. Consequently, carbimazole was temporarily withheld for three days and subsequently reintroduced at the same dosage. Repeat blood counts after 72 hours showed a TLC of 3,423 cells/mm³ with 50% neutrophils.

The patient gradually improved under the coordinated care of the Departments of Medicine, Endocrinology, Rheumatology, and Hematology. She was discharged in a clinically stable condition with a hemoglobin level of 9.6 g/dL, TLC of 4,105 cells/mm³ with 51% neutrophils, and a platelet count of 78,000/mm³. Management was primarily directed toward Graves’ disease and its associated hematological manifestations. Her discharge medications included carbimazole 10 mg twice daily, propranolol 40 mg once daily, calcium supplementation 500 mg once daily, prednisolone 20 mg twice daily, and eltrombopag 50 mg once daily. A final diagnosis of autoimmune conversion of Hashimoto’s thyroiditis to Graves’ disease, occurring in association with seropositive rheumatoid arthritis and probably immune-mediated pancytopenia, was established.

At the two-month follow-up, the patient reported significant symptomatic improvement. Her hemoglobin was 9.5 g/dL, the total leukocyte count was 4,420 cells/mm³ with 58% neutrophils, and the platelet count was stable at 75,000/mm³. Liver function tests revealed normalized liver enzymes. Thyroid function testing showed a suppressed TSH level of 0.02 mIU/L and a free T4 level of 1.8 ng/mL.

## Discussion

The present case is remarkable for the coexistence of three uncommon but immunologically linked phenomena: (1) autoimmune conversion of Hashimoto’s thyroiditis to Graves’ disease, (2) severe pancytopenia due to immune-mediated bone marrow suppression, and (3) underlying seropositive rheumatoid arthritis (RA). The concurrence of these conditions created a significant diagnostic and therapeutic challenge, particularly in the selection of antithyroid therapy in the setting of pre-existing cytopenias and deranged liver function tests.

Hashimoto’s thyroiditis (HT) and Graves’ disease (GD) represent two ends of the spectrum of autoimmune thyroid disease. The evolution of Graves’ disease into Hashimoto’s thyroiditis has been reported in about 15%-20% of cases, especially after exposure to antithyroid drug therapy. However, the reverse transition from HT to GD is distinctly uncommon [3]. A review of the published literature identified approximately 62 reported cases worldwide of conversion from HT to GD [1,3].

The “autoimmune switching” between Hashimoto’s thyroiditis (HT) and Graves’ disease (GD) probably occurs due to dynamic changes in thyroid autoimmunity and reflects a shift in the balance between stimulatory and inhibitory TSH receptor antibodies (TRAb) and immune responses (particularly Th1/Th2 cytokine activity of T helper cells), although multiple mechanisms are likely to be involved [3-5].

It may occur as early as a few months or years after the diagnosis of autoimmune hypothyroidism [4]. Patients requiring repeated reductions in levothyroxine dosage due to unexplained suppression of TSH may represent an early manifestation of this transition [1]. Our patient demonstrated several classical features suggestive of this autoimmune switch. She had documented hypothyroidism treated with levothyroxine for several years, followed by progressive suppression of TSH despite stepwise reduction and eventual discontinuation of replacement therapy. Subsequently, she developed overt thyrotoxicosis with suppressed TSH, elevated thyroid hormone levels, diffuse goiter, increased vascularity on Doppler ultrasonography, diffusely increased radionuclide uptake, and markedly elevated TRAb titers, all of which confirmed Graves’ disease. Similar clinical evolution has been reported in several case series [4,6], highlighting the need to consider Graves’ disease in patients with previous Hashimoto’s thyroiditis who develop persistent thyrotoxicosis despite withdrawal of levothyroxine.

The association between autoimmune thyroid disease and rheumatoid arthritis is well recognized. A bidirectional Mendelian randomization study by Wu et al. demonstrated evidence of a causal association between Graves’ disease and rheumatoid arthritis, highlighting a shared autoimmune genetic background [2]. The study found that a genetic predisposition to RA was associated with a 39% increased risk of developing GD, whereas genetic susceptibility to GD was associated with a 30% increased risk of developing RA. Several genetic factors have been implicated in the shared pathogenesis of these disorders, including the HLA-DRB1*16:02 allele, the cluster of differentiation 40 (CD40) gene, the insulin-like growth factor 1 receptor (IGF-1R) gene, and the non-HLA locus protein tyrosine phosphatase non-receptor type 22 (PTPN22) [2].

Hematological abnormalities are relatively common in thyrotoxicosis, with anemia reported in 10%-34% of patients, leukopenia in 15%-30%, and thrombocytopenia in 2%-5% of cases [7]. However, pancytopenia is rarely reported, with 32 cases being published in the literature worldwide [7]. It often resolves with the resolution of the hyperthyroid state. Several mechanisms have been proposed, including ineffective hematopoiesis due to excess thyroid hormone, immune-mediated destruction of circulating blood cells, hypersplenism, and direct suppression of bone marrow progenitor cells. Bone marrow findings reported in the literature are variable and include hypercellular, normocellular, and hypocellular marrows, suggesting that multiple pathogenic mechanisms may be involved.

Pancytopenia has been reported in approximately 1%-3% of patients with rheumatoid arthritis and may result from large granular lymphocyte (LGL) leukemia, autoimmune cytopenias, hemophagocytic lymphohistiocytosis (HLH), unrelated bone marrow disorders, drug-induced myelosuppression (particularly methotrexate), or coexisting autoimmune diseases such as Graves’ disease, systemic lupus erythematosus, and Sjögren’s syndrome [8,9]. Bone marrow examination showed no evidence of LGL leukemia, granulomas, HLH, or dysplasia. However, a confirmatory test to rule out LGL leukemia was not done.

Methotrexate-induced myelosuppression was also considered as a potential cause of the pancytopenia. Serial hematological data revealed that pancytopenia did not occur while the patient was receiving the drug, although there was a declining trend. Hematological parameters deteriorated following methotrexate discontinuation, with the lowest counts occurring approximately six months after withdrawal. This chronology argues against methotrexate-induced myelosuppression as the sole cause of pancytopenia, although a contributory role cannot be completely excluded. Therefore, the pancytopenia observed in this case was attributed predominantly to autoimmune origin.

Felty syndrome (FS) was also considered in the differential diagnosis because the patient had rheumatoid arthritis, neutropenia, and splenomegaly, which constitute the classic clinical triad of the syndrome. Patients with FS may develop anemia as a consequence of the anemia of chronic disease associated with longstanding RA. However, the bone marrow findings were not characteristic of Felty syndrome, which typically demonstrates a normocellular or hypercellular marrow with granulocytic hyperplasia, reflecting compensatory increased production of neutrophils in response to peripheral destruction or sequestration.

Another important clinical issue in our patient was the concern regarding initiation of antithyroid drugs (ATDs) in the presence of pre-existing leukopenia and deranged liver function. Carbimazole and methimazole are known, albeit rarely, to cause agranulocytosis, creating reluctance among clinicians to prescribe them in patients with baseline cytopenias. However, available evidence suggests that pancytopenia due to Graves’ disease itself is not a contraindication to ATD therapy and that restoration of euthyroidism is frequently associated with recovery of hematological parameters [10]. Nevertheless, careful monitoring is mandatory, especially in patients with severe neutropenia.

Also, propylthiouracil was avoided because of persistently elevated transaminases and the risk of hepatocellular failure, while carbimazole was started cautiously following improvement in leukocyte counts induced by G-CSF. The transient fall in TLC following the initiation of carbimazole raised concern for potential drug-related worsening; however, subsequent stabilization and clinical improvement suggested that continued treatment was feasible under close hematological surveillance. The use of G-CSF, corticosteroids, and eltrombopag likely contributed to hematological recovery, while carbimazole controlled the underlying thyrotoxicosis. Thus, the recovery of blood counts likely reflected a combination of treatment effects and control of the underlying autoimmune thyroid disease process.

We planned definitive surgical management after achieving a euthyroid state, as surgery would avoid prolonged exposure to antithyroid medication and potential concerns associated with radioactive iodine therapy. Thyroidectomy is expected to provide definitive control of hyperthyroidism and reduce the risk of relapse.

## Conclusions

This case highlights a rare autoimmune transition from Hashimoto’s thyroiditis to Graves’ disease occurring in a patient with seropositive rheumatoid arthritis and immune-mediated pancytopenia. The presence of RA in a patient with HT and hypothyroidism should alert physicians to monitor thyroid function on a periodic basis, not only for euthyroid status but also for conversion to Graves’ disease. Furthermore, severe pancytopenia should not automatically preclude antithyroid therapy once alternative causes have been excluded and appropriate hematological support is provided. A multidisciplinary approach involving endocrinology, hematology, rheumatology, and internal medicine is essential for achieving favorable outcomes in such complex presentations.
